# Isolation, toxin gene profiling, and phylogenetic analysis of *Clostridium perfringens* in Egyptian fruit bats: public health and epidemiological implications

**DOI:** 10.1038/s41598-025-26288-3

**Published:** 2025-11-18

**Authors:** Toka A. Allam, Fatma Abdel-kader, Mona Kadry

**Affiliations:** Department of Zoonoses, Faculty of Veterinary Medicine, Cairo University, PO Box 12211, Giza, Egypt

**Keywords:** Bat, *Clostridium perfringens*, Toxin genes, Genotyping, Egypt, Ecology, Ecology, Genetics, Microbiology, Molecular biology, Zoology

## Abstract

*Clostridium perfringens* (*C. perfringens*) is spore forming, toxin producing bacterium causing serious diseases in both animals and man and its presence in bats, especially the Egyptian fruit bat, are ecologically important yet increasingly interact with human environments due to habitat changes which raise the concerns about their role as reservoirs for zoonotic pathogens. This study, the first of its kind in Egypt, investigates the occurrence and characteristics of *C. perfringens* in bats to evaluate their potential role as reservoirs for this toxin-producing, environmentally persistent foodborne pathogen. Fifty fruit bats were captured using mist nets at foraging and roosting sites. The bats were identified morphologically, and for each bat, fecal swabs and internal organs were collected (n = 100). The samples were examined bacteriologically to investigate the *C. perfringens* detection then confirmed biochemically and via gram staining. DNA was extracted, and toxin genotyping was conducted using multiplex PCR for main toxin genes “ *cpa*,* cpb*,* etx*,* ia*,* netB*,* cpe*” whereas uniplex PCR for *cpb2*. Sequencing and phylogenetic analysis of *cpb2* gene from four isolates were analyzed to determine genetic relatedness. Out of 100 samples examined, *C. perfringens* was detected in 31% (31/100) of samples, with similar occurrence in internal organs (30%) and fecal swabs (32%). All isolates carried the *cpa* gene (100%), while *cpb*, *cpe*, and *cpb2* were detected in 83.9%, 64.5%, and 64.5% of isolates, respectively; *ia*,* etx*,* netB* genes were not detected. Notably, 35.5% of isolates harbored both *cpe* and *cpb2* genes. Toxinotyping showed type C as predominant (83.9%), followed by type F (12.9%) and type A (3.2%), highlighting the epidemiological significance of type C strains. Phylogenetic analysis of *cpb2* sequences indicated high genetic similarity among bat isolates and close relationships with strains from domestic animals and environmental sources, suggesting possible shared habitats and horizontal gene transfer. These findings identify bats as potential reservoirs of toxigenic *C. perfringens*, reinforcing the importance of integrating wildlife into One Health surveillance strategies. This study reports the first detection of *C. perfringens* from Egyptian fruit bats. Phylogenetic analysis revealed close genetic links to strains from domestic animals and environmental sources and these findings highlight bats’ potential role as reservoirs of virulent *C. perfringens.*

## Introduction

Recent pandemics have increased global concern over zoonotic diseases, emphasizing the impact of anthropogenic environmental changes on increasing interactions between humans and wildlife, which in turn facilitates the transmission of infection from animals to humans^1,2^. Among wildlife species, bats (order Chiroptera) are of particular significance due to their exceptional biological and ecological characteristics and the only mammals capable of constant flying, bats exhibit high species richness with over 1,300 identified species comprising nearly 20% of all known mammals and display broad geographical distribution, diverse life history traits, and varied dietary habits, these factors, coupled with their ability to host and tolerate numerous pathogens without manifesting disease symptoms, position bats as critical natural reservoirs for several emerging infectious diseases^3,4^.

Human-driven activities such as deforestation, urbanization, and habitat fragmentation increase human–bat interactions and promote zoonotic spillover^2^. These environmental changes alter bat roosting and foraging behaviors, enhancing the risk of indirect transmission through contamination of soil, water, or food with bat excreta^5,6^.

The fruit bat (*Rousettus aegyptiacus*) is considered the most widespread and ecologically significant fruit bat species in Egypt found throughout the Nile Valley and in several oases, plays an essential ecological role in seed dispersal and pollination^7,8^ .However, its behavior particularly its large colony size, frequent presence in agricultural settings, and habit of chewing and discarding fruits brings it into close contact with human environments, heightening concern regarding its potential role in zoonotic diseases transmission^9^.

After COVID 19 pandemic, bats attracted the world as reservoirs of emerging viruses with the potential to cause severe infections in humans^10^, but the limited research on bacterial infections in bats and their potential impact on public health leaves significant gaps in understanding their contribution to zoonotic diseases and they are recognized as potential carriers of various foodborne pathogens which have been particularly detected in their feces, suggesting a possible role in environmental contamination and disease transmission while their ability to fly may facilitate the spread of these pathogens to crops or livestock and human^3,11,12^.

*C. perfringens*, a Gram-positive spore-forming anaerobe, is recognized as an important pathogen in human and animal infections^13^. The heat and oxygen resistance of its spores, enabling the bacterium to survive in environments ranging from soil and sewage to feces and host gastrointestinal tracts. It is recognized globally as a major foodborne pathogen of significant public health concern^14^. The organism’s pathogenicity and virulence are largely driven by its capacity to produce a broad range of toxins, which are crucial in the pathogenesis of serious diseases affecting both humans and animals^15^.

In 2018, *C. perfringens* strains were reclassified into seven types, (A_G) based on the presence of six principal toxins: alpha (α), beta (β), epsilon (ε), iota (ι), enterotoxin (CPE), and necrotic enteritis B-like toxin (NetB). Each type is defined by its distinct toxin profile^16^. Beyond these major toxins, it also produces accessory toxins, including perfringolysin O (theta toxin) and beta-2 toxin (CPB2). Although not part of the toxinotyping scheme, accessory toxins contribute to pathogenicity by interacting with extracellular toxins, modulating their production and virulence, and impacting the severity and progression of disease^17^.

Among the toxinotypes, Type A characterized by the production of α-toxin is frequently associated with human gas gangrene and is also implicated in foodborne illnesses and antibiotic-associated diarrhea^15^. Type C (that produce α and β toxins) is responsible for enterotoxaemia, serious conditions affecting various mammals and humans and for pigbel, a severe necrotizing enteritis in humans^17^. Type F strains defined by its expression of α-toxin and CPE are important causes of foodborne and non-foodborne intestinal infections in man, including antibiotic-associated diarrhea and occasional diarrhea^14^.

Type B, producing α, β, and ε toxins, is linked to hemorrhagic enteritis in young livestock, such as foals, calves, sheep, also lamb dysentery. Type D, which expresses α and ε toxins, causes pulpy kidney disease in lambs and may also affect goats and cattle. Type E, associated with α and ι toxins, is implicated in enterotoxemia in calves and lambs. Type G, primarily responsible for necrotic enteritis in poultry, was recently redefined to include strains co-producing NetB and CPE toxins^14,18,19^.

In Egypt, recent research has focused on detecting *C. perfringens* among cattle, buffaloes, sheep, goats, camels, and poultry^20–23^, However, this is the first study on bats which highlights a valuable opportunity for us for investigation. Therefore, this study aimed to detect and characterize *C. perfringens* in Egyptian fruit bats, determine the distribution of its toxin genes, and evaluate its potential epidemiological significance.

## Materials and methods

### Bat Capture, identification and sample collection

In this study, fifty fruit bats were captured using mist nets strategically placed at foraging and roosting sites. At the time of euthanasia, by an overdose of isoflurane, their body weight ranged from 100 to 130 g. Species identification was performed based on external morphological features, following the taxonomic keys of Dietz^24^ and Monadjem et al.^25^, diagnostic traits observed included, large eyes and simple ears without tragus or antitragus, which are characteristic of the family Pteropodidae. The presence of claws on both the first and second digits further confirmed this classification. Morphometric measurements such as Wingspan, forearm length, and tail length were taken using a mechanical caliper, these morphological criteria collectively confirmed that all specimens belonged to the genus Rousettus. From each bat, two types of biological samples were collected aseptically: fecal swabs and internal organs. In total, 100 samples were obtained, comprising 50 fecal swabs and 50 internal organs samples.

### Isolation and identification of *Clostridium perfringens*

Each sample was aseptically enriched into sterile, freshly prepared Cooked Meat Medium (HiMedia, India) then incubated anaerobically at 37 °C for 24 to 48 h using an anaerobic jar with a gas-generating kit (HiMedia, India). After enrichment, a loopful of the culture was plating on tryptose sulfite cycloserine (TSC) agar (HiMedia, India), supplemented with 5% egg yolk emulsion and *Clostridium perfringens* selective supplement (HiMedia, India), and incubated anaerobically at 37 °C for 24 h, as described by Kotsanas et al.^26^. colonies exhibiting black pigmentation and surrounded by an opaque halo were presumptively identified as *C. perfringens*, attributed to lecithinase activity. These presumptive colonies were subcultured to obtain pure isolates, which were subsequently examined microscopically following Gram staining. The isolates appeared as large, Gram-positive bacilli consistent with the morphology of *C. perfringens*. Biochemical identification was accomplished according to Procop et al.^27^.

To maintain biosafety and prevent laboratory contamination, standard precautions were rigorously implemented. These included the usage of personal protective equipment (PPE), strict hand hygiene practices, routine disinfection of work surfaces and instruments, and the proper disposal of biological waste materials.

### Extraction of the genomic DNA

Genomic DNA was extracted from all isolates using the boiling method as described by Rana et al.^28^, the Nanodrop spectrophotometer was used to check each sample’s DNA concentration and purity then the DNA was stored at − 20 °C until further use.

### Toxin gene profiling of *Clostridium perfringens* isolates

Multiplex polymerase chain reaction (PCR) was employed to genotype all 31 confirmed *C. perfringens* isolates by amplifying genes encoding alpha-toxin (*cpa)*, beta-toxin (*cpb*), epsilon-toxin (*etx*), *iota*-toxin (i.a.), necrotic enteritis B-like toxin (*netB*), and enterotoxin (*cpe*), following the protocols described by Hamza et al.^29^ and Rood et al.^16^. Detection of beta2-toxin gene (*cpb2*) was carried out separately using a conventional uniplex PCR assay as outlined by Yang et al.^30^. Each PCR reaction mixture (25 µL) contained 5 µL of template DNA, 12.5 µL of 2X amaR OnePCR™ (GeneDireX, Inc., USA), 1 µL of each primer (10 pmol/µL; Metabion, Germany), and 5.5 µL of PCR-grade water. Additionally, PCR reaction was done using *C. perfringens* (ATCC 13124) as a positive control, while water sample was used as a negative control. The amplification conditions included an initial denaturation at 94 °C for 5 min, followed by 35 cycles of denaturation at 94 °C for 1 min, annealing at 55 °C for 1 min, and extension at 72 °C for 1 min, with a final extension at 72 °C for 10 min. PCR products were separated by electrophoresis on 1.5% agarose gels prepared in 0.5× Tris-borate-EDTA (TBE) buffer, and DNA bands were visualized under Ultraviolet light. The sequences of the primers used for toxin gene detection in *C. perfringens* isolates along with their expected amplicon sizes, are presented in Table 1.

**Table 1:** Primers with specific sequence and amplicon size used for toxin gene detection in *Clostridium perfringens* isolates.

Toxin gene		Primer sequence	Amplicon size (bp)	Reference
*cpa*	*cpa F*	GCTAATGTTACTGCCGTTGA	*324*	^29^
	*cpa R*	CCTCTGATACATCGTGTAAG		
*cpb*	*cpb F*	GCGAATATGCTGAATCATCTA	*196*	^29^
	*cpb R*	GCAGGAACATTAGTATATCTTC		
*etx*	*etx F*	GCGGTGATATCCATCTATTC	*655*	^29^
	*etx R*	CCACTTACTTGTCCTACTAAC		
i.a.	*i.a. F*	ACTACTCTCAGACAAGACAG	*466*	^29^
	*i.a. R*	CTTTCCTTCTATTACTATACG		
*net B*	*net B F*	CTTCTAGTGATACCGCTTCAC	*738*	^16^
	*net B R*	CGTTATATTCACTTGTTGACGAAAG		
*cpe*	*cpe F*	ACTACTCTCAGACAAGACAG	*233*	^29^
	*cpe R*	CTTTCCTTCTATTACTATACG		
*cpb2*	*cpb2F*	AGATTTTAAATATGATCCTAACC	*567*	^30^
	*cpb2R*	CAATACCCTTCACCAAATACTC		

### Sequencing and phylogeny

PCR products of the *cpb2* gene obtained from four randomly selected *C. perfringens* isolates (two derived from fecal samples and two from bat internal organs) were purified using the GeneJET PCR Purification Kit (Thermo Scientific) according to the manufacturer’s protocol. Sequencing was carried out on a DNA sequencer with the BigDye Terminator v3.1 Cycle Sequencing Kit (Applied Biosystems). The resulting sequences were aligned with reference sequences in the GenBank database of the National Center for Biotechnology Information (NCBI) using the BLAST tool. Phylogenetic analysis was performed to evaluate the genetic relatedness of the *cpb2* sequences from different tissue origins. Sequence alignment and identity matrices were generated with the ClustalW multiple alignment tool in BioEdit, and phylogenetic trees were constructed using the maximum likelihood approach in MEGA6 (version 6.06).

### Statistical analysis

Statistical analyses were performed using R (version 4.4.0). Isolates classification was carried out with the pheatmap package (version 1.0.13).

## Results

### The occurrence of *Clostridium perfringens* in different sample types

**Fig. 1:**
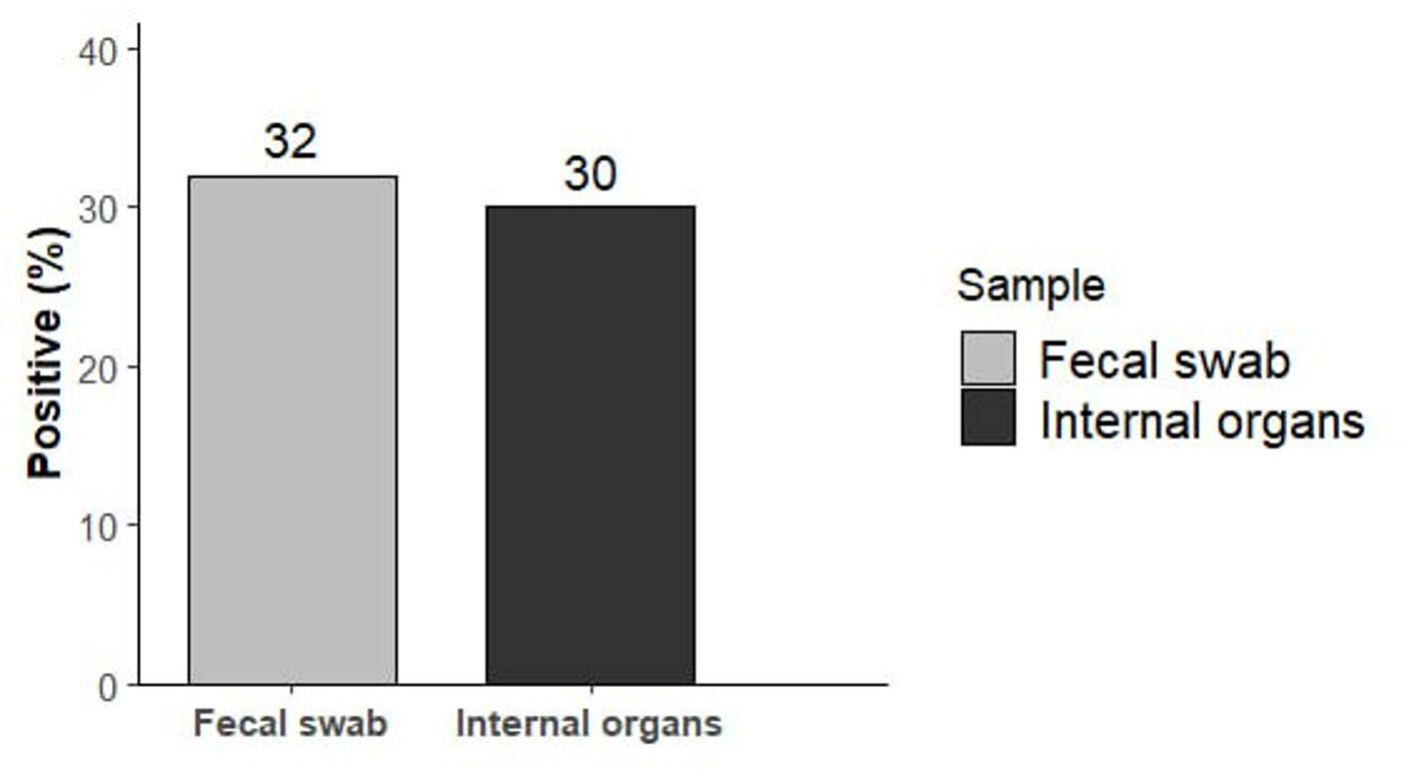
Occurrence of *C. perfringens* by sample Type.

A total of 100 samples equally divided between internal organs and fecal swabs (50 each) were examined for *C. perfringens* isolation. As illustrated in Fig. 1, out of the 50 internal organs samples analyzed, 15 tested positives for *C. perfringens*, yielding an occurrence of 30% while of the 50 fecal swab samples, 16 were positive, corresponding to a slightly higher occurrence of 32%. Overall occurrence combining both sample types, *C. perfringens*, was detected in 31% (31/100) of the samples.

### Frequency of toxin gene profiles in *Clostridium perfringens* isolates

**Fig. 2:**
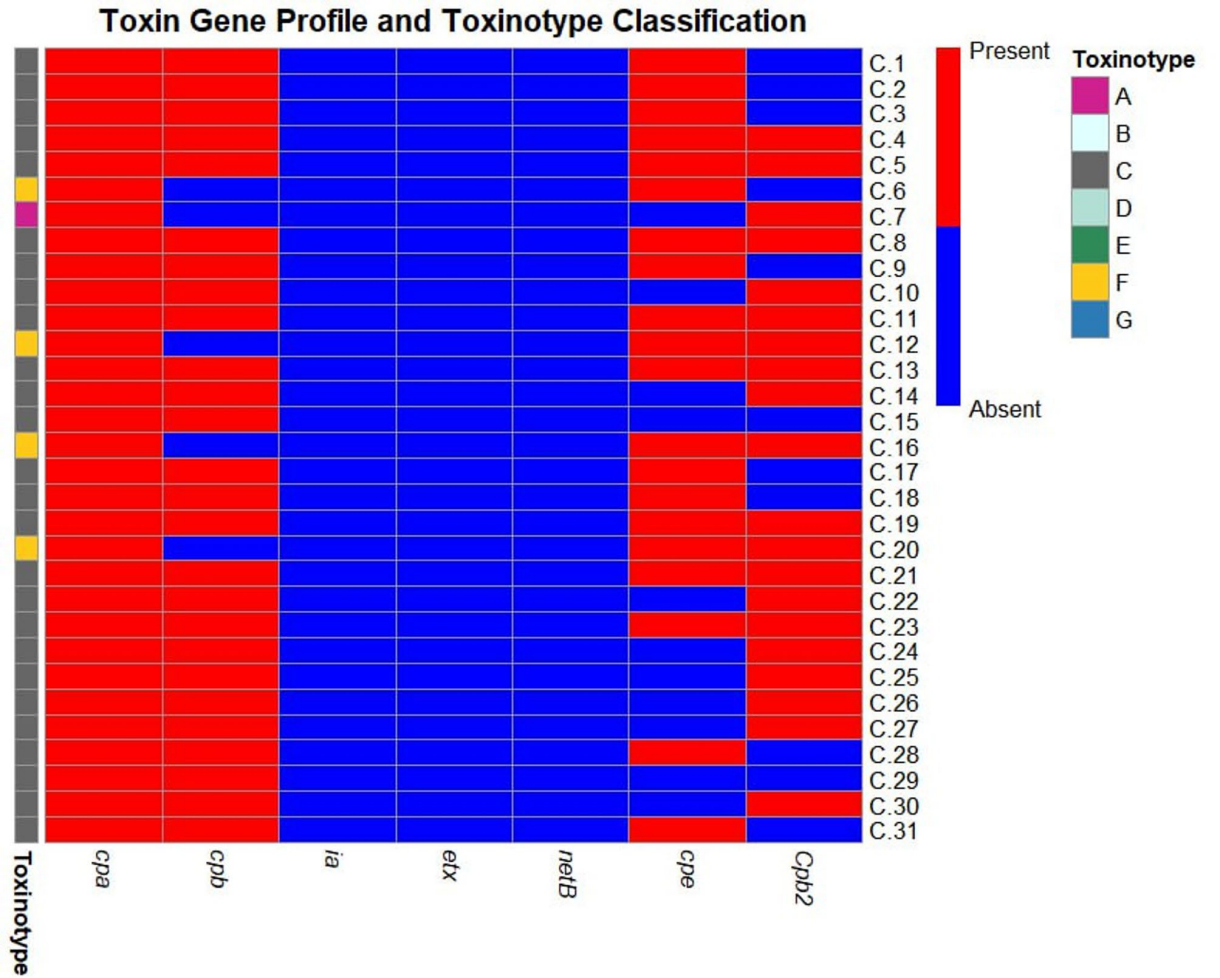
Heatmap of *C.perfringens* isolates categorized by toxin gene profiles (*cpa*,* cpb*, i.a.,* etx*,* netB*,* cpe*, and *cpb2*) based on the updated toxintype scheme of Rood et al.^16^. , with the *cpb2* gene included as an additional virulence marker. Color gradients indicate the presence (red) or absence (blue) of genes across isolates, with toxinotypes A–G annotated on the left.

As in Table 2; Fig. 2 in this study, analysis the toxin gene profiles of the 31 *C. perfringens* isolates revealed that all carried the *cpa* gene (100%) while the *cpb* gene was detected in 26/31 isolates (83.9%) where 14/15 (93.3%) internal organs isolates and 12/16 (75.0%) fecal swab isolates while i.a.,* etx*,* netB* genes were not detected in isolates 0 (0%). The *cpe* gene was present in 8/15 (53.3%) internal organs isolate and 12/16 (75.0%) fecal swab isolates, giving an overall occurrence of 64.5% (20/31). The *cpb2* gene was identified on 8/15 (53.3%) internal organs isolate and 12/16 (75.0%) fecal swab isolates, with a total occurrence of 64.5% (20/31). Notably, 35.5% (11/31) of isolates carried both *cpe* and *cpb2* genes.

**Table 2:** Frequency of toxin genes in *C. perfringens* isolates.

Toxin gene	Internal organs isolates (*n*, %)	fecal Swab isolates (*n*, %)	Total isolates (*n*, %)
*cpa*	15 (100%)	16 (100%)	31 (100%)
*cpb*	14 (93.3%)	12 (75%)	26 (83.9%)
i.a.	0(0%)	0(0%)	0(0%)
*etx*	0 (0%)	0(0%)	0(0%)
*netB*	0 (0%)	0(0%)	0 (0%)
*cpe*	8 (53.3%)	12 (75%)	20 (64.5%)
*cpb2*	8 (53.3%)	12 (75%)	20 (64.5%)

### Toxinotyping distribution of *Clostridium perfringens* isolates

**Fig. 3:**
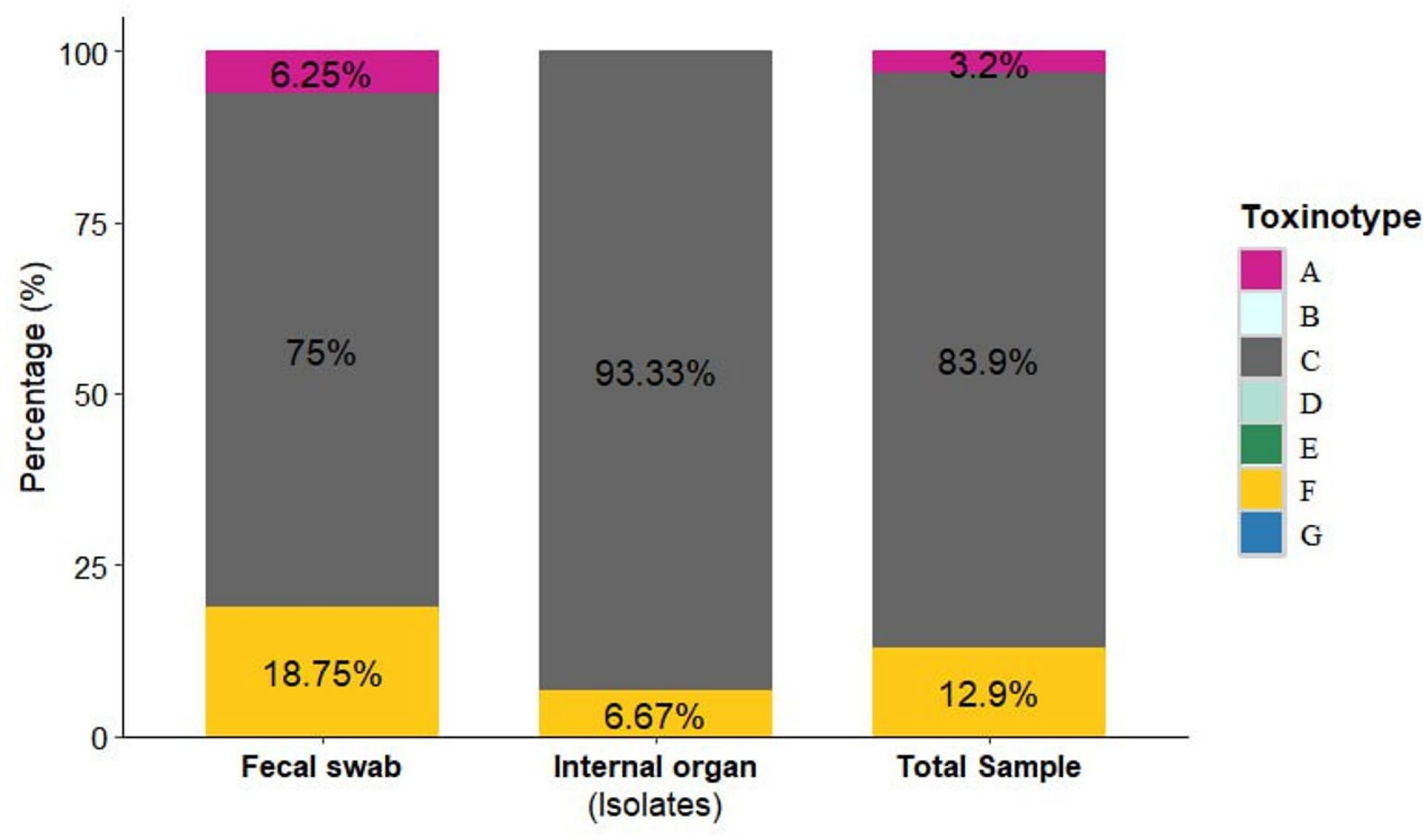
Comparative distribution of *C. perfringens* toxinotypes among 31 isolates obtained from internal organs (*n* = 15) and fecal swabs (*n* = 16) of Egyptian fruit bats. Type C predominated in both sample types (93.33% in internal organs, 75% in fecal swabs), followed by Type F (6.67% and 18.75%, respectively) and Type A (6.25% only in fecal swabs). Overall, Type C represented 83.9% of positive total sample isolates (*n* = 31), followed by Type F (12.9%) and Type A (3.2%). No isolates of Types B, D, E, or G were detected (0%).

As shown in Fig. 3 of the 31 *C. perfringens* positive isolates, toxinotyping revealed that type C was the most frequent, detected in 14/15 (93.33%) of the internal organs isolates and 12 /16 (75.0%) of the fecal swab isolates. Type F was identified in 1/15 (6.67%) internal organ isolates and 3/16 (18.75%) fecal swab isolates, while type A was detected only in 1/16 (6.25%) fecal swab isolate. Overall, type C accounted for 83.9% of all positive samples, followed by type F (12.9%) and type A (3.2%), where type B, D, E, G not detected (0%).

## Discussion

Among mammals, bats are distinguished by their capacity to migrate over a prolonged distances accordingly there is a growing concern regarding their role as potential carriers and transmitters of zoonotic diseases^31^. They are recognized as potential carriers of various foodborne pathogens, including *Salmonella*^32,33^, *Shigella*^34^, *Yersiina*^35^, *Campylobacter*^36,37^, the detection of bat feces carrying these bacteria may act as a source of environmental and health hazards. In this study *C. perfringens*, a well-recognized etiological agent of foodborne illness, was detected in 32% of fecal swabs and 30% of internal organs samples in bats (Fig. 1**)**, with an overall occurrence rate of 31% and this agrees with Hajkova et al.^38^ and with Henry et al.^39^ who reported *C. perfringens* previously in bats. It has been proven that *C. perfringens* is responsible for various gastrointestinal illnesses in humans and animals^20^ and the systemic absorption of its intestinal toxins can cause enterotoxemia^40^.

This study highlights the potential zoonotic risk associated with bats, as *C. perfringens* can be excreted in feces and, in some cases, proliferate in tissues, leading to severe infections. The presence of bacterial spores in animal waste or decaying tissues may contribute to environmental contamination and increase opportunities for indirect transmission to humans and other animals^41^.

The pathogenicity of *C. perfringens* is intervened by one or more of its powerful toxin genes^40^. In the current study, molecular characterization of 31 *C. perfringens* isolates obtained from bats revealed the presence of several toxin genes, particularly *cpa*,* cpb*,* cpe*, and *cpb2*, where all isolate carried the *cpa* gene (alpha toxin gene), that commonly used for the identification of this bacterium^16^, and this align with previous studies by Abo Elyazeed et al.^42^, and Li et al.^43^ which revealed that the *cpa* gene present in all *C. perfringens* isolates and it is consistent with its essential role in their virulence by enzymatically targeting cell membranes causing damage across many animal species and culminating in cell lysis and hemolysis^44–47^.

In our study, the *cpb* gene was found in 83.9% of isolates, a finding of considerable public health and epidemiological significance. This gene encodes for *C. perfringens* beta toxin, a toxin and a key virulence factor of *C. perfringens* type C^48^, which are strongly related to severe enteritis in man and animals by targeting both intestinal epithelial cells and endothelial cells^49^ whereas the *cpe* gene was present in the study in 64.5% of the isolates, with 16 isolates classified as CPE-positive type C strains that have drawn attention due to the presence of the *cpe* gene, which has been suggested as a possible contributor to the development of pigbel disease in humans^50^ and 4 isolates classified as type F with new the updated scheme of Rood et al.^16^ This enterotoxin gene is the key toxin responsible for food born intoxication and other intestinal conditions in man^51^ Furthermore, case reports^52–54^ suggest that it may be responsible for disease in domestic as well as wild animals.

The *cpb2* gene was identified in our isolates with a total occurrence of 64.5% classified as 3 isolates of type F, one of type A and 16 of type C, the elevated prevalence of *cpb2*-positive strains in our study support the role of beta2 toxin as a key virulence factor in enteric infections, aligns with the findings of Bueschel et al.^55^ who reported a high frequency of *cpb2* among *C. perfringens* isolates including animal and environmental samples. Over the past decade *cpb2* gene has been associated with a possible role in enteric diseases affecting a wide range of food-producing and wild animals^56–58^ and human^59,60^ Although it is not used for toxinotyping and its significance in many species remains unclear, its presence has been associated with cases of diarrhea in animals and may enhance virulence in co-infection scenarios^61^.

The notable co-occurrence of *cpe* and *cpb2* genes in 35.5% of *C. perfringens* isolates in our study may be attributed to potential genetic linkage. Li et al.^49^ reported that these genes can be plasmid-borne, either jointly or independently, while Fisher et al.^60^ observed that *C. perfringens* type A strains associated with antibiotic-associated diarrhea and sporadic diarrhea frequently harbor both genes on the same plasmid. This shared genetic localization could facilitate their simultaneous expression, thereby amplifying pathogenic potential through complementary mechanisms where *cpe* disrupting epithelial tight junctions and *cpb2* enhancing cytotoxic effects. Such co-expression may contribute to more severe disease outcomes^60,62^. Beyond genetic factors, ecological influences may also play a role. The bat gastrointestinal ecosystem characterized by unique dietary components and close social interactions within roosts may promote the persistence and horizontal transfer of plasmids carrying these toxin genes, favoring the emergence of dual-positive *C. perfringens* strains^63,64^.

The isolates in this study exhibited considerable heterogeneity and were classified into multiple toxinotypes, namely types A, C, and F. Notably, type C was the most prevalent, representing 83.9% of all positive samples. This type was detected in 93.33% of internal organs isolates and 75.0% of fecal swab isolates, indicating its broad distribution across both internal and excretory tissues in bats. The predominance of type C is epidemiologically significant, as it produces both α- and β - toxins and has been strongly associated with severe enteric diseases, including hemorrhagic and necrotic enteritis, particularly among neonates and immunocompromised individuals^40^. These findings support the hypothesis that *C. perfringens* type C may successfully colonize or infect the intestinal and renal systems of bats, reinforcing concerns over their role as potential reservoirs. Moreover, its high occurrence supports the argument by Johnson et al.^65^ who proposed that animal-to-human transmission is more plausible in cases involving enterotoxigenic *C. perfringens* foodborne illness, particularly those caused by type C strains. The presence of these virulent strains in bats point out the risk of zoonoses and calls for further investigation into their role in public health.

Most *C. perfringens* foodborne outbreaks have been linked to what are now known as type F isolates^14,66^. In the current study, *C. perfringens* type F was detected in 12.9% of the isolates. Although less prevalent than type C, the presence of type F is noteworthy due to its known association with enteric diseases such as food poisoning and antibiotic-associated diarrhea in humans^67^. The detection of this toxinotype in bats highlights their potential role as asymptomatic carriers. This aligns with findings from recent genomic study in Ghana where type F isolates were retrieved from clinical wastewater samples^68^ that emphasize the pathogenic potential of type F strains harboring the *cpe* gene, even when isolated from non-human or environmental sources reinforcing the need to consider bats as possible environmental reservoirs of zoonotic *C. perfringens* strains.

Finding type F in Ghana and Egypt, two African countries, emphasizes the necessity of extending *C. perfringens* molecular surveillance beyond clinical settings to include environmental and animal sources. The ecological behavior of bats such as their extensive movement and roosting close to human populations, may promote the spread of pathogenic strains geographically and contaminate the environment. According to our results, bats may serve as environmental reservoirs for enterotoxigenic *C. perfringens* type F, which could pose a risk to public health through indirect transmission.

In the present study, *C.perfringens* type A was detected in one isolate obtained from bat fecal samples in Egypt. The detection of *C. perfringens* type A harboring beta2 toxin gene is particularly noteworthy, as Kiu et al.^48^ previously linked a similar strain to a human life-threatening case of preterm necrotizing entercolitis. Type A strains can cause disease in various mammalian species, either independently or in association with other Clostridium species^49^. Their pathogenicity is primarily linked to α-toxin, and although they represent only a small proportion of all isolates, their detection remains of epidemiological significance. This is supported by a recent genomic studies which found that type A isolates have extra virulence genes which are linked to increased cytotoxicity and gastrointestinal environment adaptation^17,68^. These findings highlight the pathogenic potential of type A strains and emphasize the need for continued surveillance, as even low-frequency detection in wildlife reservoirs like bats could pose future zoonotic or environmental risks.

**Fig. 4:**
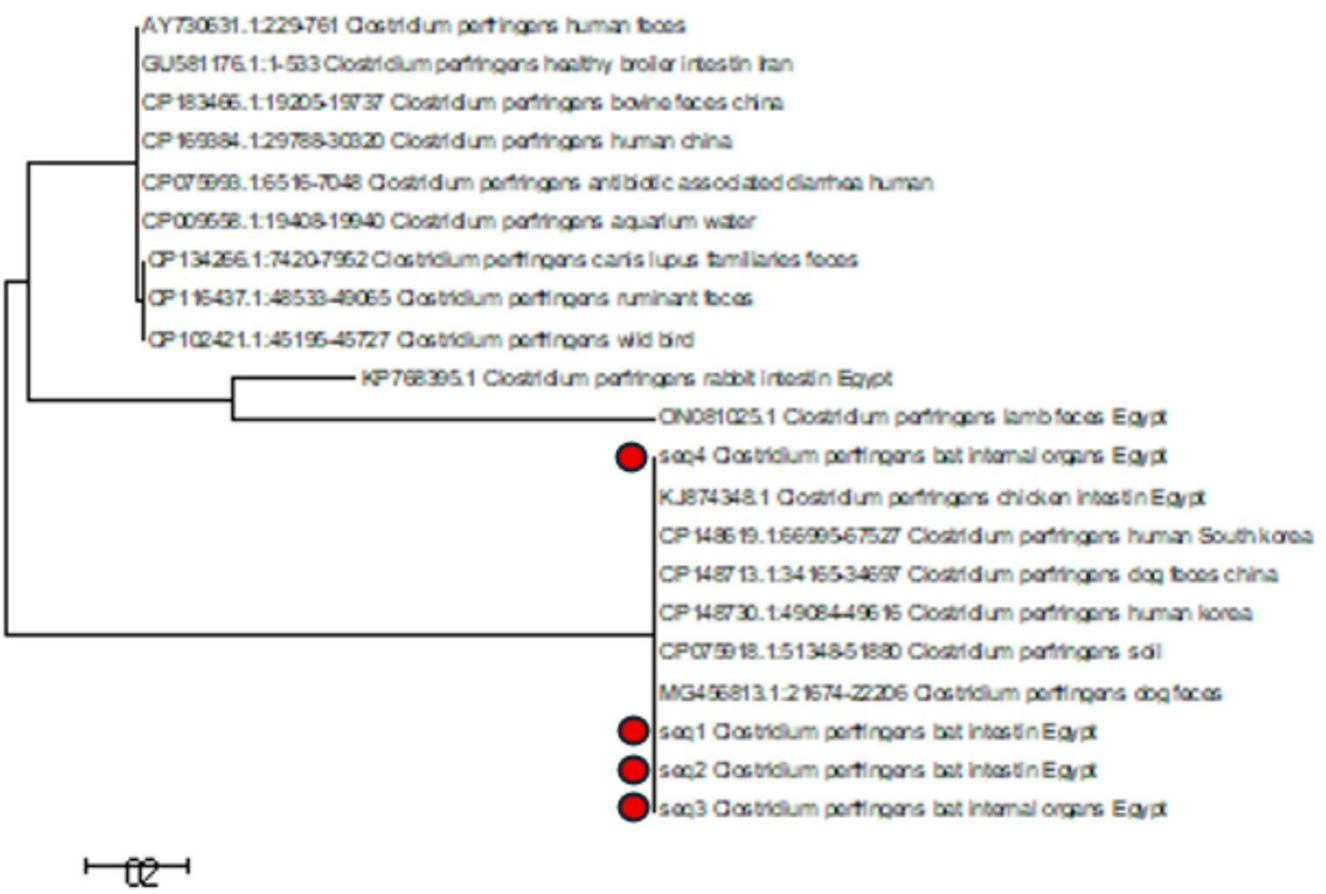
A maximum likelihood phylogenetic tree was composed of MEGA6 software (version 6.06). Using nucleotide sequences of four *cpb2* genes from *C. perfringens* isolates. The isolates in this study were taken from fecal swabs and internal organs samples.

The phylogenetic analysis (Fig. 4) of the *C. perfringens cpb2* gene sequences (accession numbers: PX233843, PX381350, PX254367 and PX381349) revealed distinct clustering patterns that reflect both the genetic diversity and potential epidemiological connections among isolates from different sources. Notably, the four sequences isolated from bats (seq1–seq4), derived from fecal swabs and internal organs samples, clustered closely together, forming a well-supported sub-clade. This tight clustering indicates a high level of genetic similarity among the bat isolates, suggesting a common source or shared evolutionary origin.

It’s interesting to note that the sequences derived from bats were closely related to isolates from Egypt, especially those derived from lamb feces and chicken intestines as in Fig. 4. Because of their intimate relationship, bats and domestic animals may have ecological or environmental connections, suggesting shared habitats, feeding areas, or exposure to common contaminated resources^69,70^.

Additionally, isolation from environmental sources such soil and human and dog feces were included in this sub-clade. Given that many of these *cpb2* genes are plasmid-associated, the existence of several host origins within the same phylogenetic group raises the possibility of horizontal gene transfer^60^. Such plasmids’ mobility may make it easier for the *cpb2* gene to spread throughout many hosts and surroundings, which would be extremely concerning for public health. Also, Bat-associated strains are genetically distinct from human clinical strains (e.g., diarrhea-related strains from Finland and China), however the close phylogenetic relationship among *cpb2*-positive isolates from different sources suggests the gene is widespread and may transmit between species.

The detection of *cpb2* gene in bat isolates indicate that bats may serve as a reservoir for toxigenic *C. perfringens*. Their interactions within both natural and anthropogenic environments emphasize the importance of investigating their role in *C. perfringens* epidemiology, particularly within the One Health framework that links human, animal, and environmental health. However, this study is limited by its relatively small sample size and the lack of antimicrobial susceptibility testing, which would have provided deeper insight into the potential public health implications.

## Conclusion

This study provides the first evidence of *C. perfringens* occurrence and toxin gene diversity in Egyptian fruit bats. The predominance of type C, along with detection of types F and A, and the high frequency of, *cpb*,* cpe*,* and cpb2* genes, highlights the potential of bats to act as reservoirs for highly virulent, toxin-producing strains. Phylogenetic analysis demonstrated genetic relatedness between bat isolates and strains from domestic animals and environmental sources, suggesting possible ecological overlap and horizontal gene transfer. Further studies are recommended to assess the virulence gene profiles and potential pathogenicity of these strains, as well as their public health relevance to better understand and mitigate zoonotic risks.

## Data Availability

All the data generated or analysed in this study are included in this published article. The nucleotide sequences generated during the current study have been deposited in the NCBI GenBank system under accession numbers PX233843– PX254367.
